# Induction of tumour necrosis factor receptor-expressing macrophages by interleukin-10 and macrophage colony-stimulating factor in rheumatoid arthritis

**DOI:** 10.1186/ar2015

**Published:** 2006-07-19

**Authors:** Koji Takasugi, Masahiro Yamamura, Mitsuhiro Iwahashi, Fumio Otsuka, Jiro Yamana, Katsue Sunahori, Masanori Kawashima, Masao Yamada, Hirofumi Makino

**Affiliations:** 1 Department of Medicine and Clinical Science, Graduate School of Medicine, Dentistry, Pharmaceutical Sciences, Okayama University, 2-5-1 Shikata-cho, Okayama 700-8558, Japan; 2 Department of Rheumatology, School of Medicine, Aichi Medical University, Nagakute-cho, Aichi 480-1195, Japan; 3 Department of Virology, Graduate School of Medicine, Dentistry, Pharmaceutical Sciences, 2-5-1 Shikata-cho, Okayama 700-8558, Japan

## Abstract

Despite its potent ability to inhibit proinflammatory cytokine synthesis, interleukin (IL)-10 has a marginal clinical effect in rheumatoid arthritis (RA) patients. Recent evidence suggests that IL-10 induces monocyte/macrophage maturation in cooperation with macrophage-colony stimulating factor (M-CSF). In the present study, we found that the inducible subunit of the IL-10 receptor (IL-10R), type 1 IL-10R (IL-10R1), was expressed at higher levels on monocytes in RA than in healthy controls, in association with disease activity, while their expression of both type 1 and 2 tumour necrosis factor receptors (TNFR1/2) was not increased. The expression of IL-10R1 but not IL-10R2 was augmented on monocytes cultured in the presence of RA synovial tissue (ST) cell culture supernatants. Cell surface expression of TNFR1/2 expression on monocytes was induced by IL-10, and more efficiently in combination with M-CSF. Two-color immunofluorescence labeling of RA ST samples showed an intensive coexpression of IL-10R1, TNFR1/2, and M-CSF receptor in CD68^+ ^lining macrophages. Adhered monocytes, after 3-day preincubation with IL-10 and M-CSF, could produce more IL-1β and IL-6 in response to TNF-α in the presence of dibutyryl cAMP, as compared with the cells preincubated with or without IL-10 or M-CSF alone. Microarray analysis of gene expression revealed that IL-10 activated various genes essential for macrophage functions, including other members of the TNFR superfamily, receptors for chemokines and growth factors, Toll-like receptors, and TNFR-associated signaling molecules. These results suggest that IL-10 may contribute to the inflammatory process by facilitating monocyte differentiation into TNF-α-responsive macrophages in the presence of M-CSF in RA.

## Introduction

Macrophages play an important role in both chronic inflammation and joint destruction in rheumatoid arthritis (RA), principally by producing many proinflammatory cytokines such as tumour necrosis factor-α (TNF-α) [1]. The significance of TNF-α in the pathogenesis has been well proven by the clinical efficacy of its blockade in RA patients with active disease [2]. The pleiotropic effects of TNF-α are mediated through two distinct TNF receptors, the type 1 p60/p55 receptor (TNFR1) and the type 2 p80/p75 receptor (TNFR2) [3,4]. TNFR1 is expressed in all cell types and activates various cellular responses through the transcription factor NF (nuclear factor)-κB and apoptosis [5-7]. In contrast, TNFR2 is expressed by cells of the immune system and endothelial cells, and its precise role is less clear [7,8]. However, TNFR2 mediates part of TNF effects, including proliferation of T cells and B cells, NF-κB activation, and cytotoxicity, and may potentiate the effects of TNFR1 by ligand passing to the lower-affinity TNFR1 [9,10].

Interleukin (IL)-10 has been recognised as a key cytokine that modulates the cell-mediated immune response by regulating activation and effector function of T cells and monocytes/macrophages, most notably by inhibiting the production of cytokines such as TNF-α, IL-1, IL-6, and interferon-γ [11]. IL-10 binds to the IL-10 receptor (IL-10R) complex composed of two subunits, the primary ligand-binding component IL-10R1 and the accessory component IL-10R2. IL-10R2 is constitutively expressed, but IL-10R1 is inducible; IL-10R1 thus seems critical in the IL-10-mediated cellular response [11]. Interestingly, TNF-α effectively induces IL-10 synthesis in monocytes, which represents the major negative feedback to its own production [12]. In the synovial tissue (ST) of RA, high levels of IL-10 are expressed in the lining layer and mononuclear cell aggregates, presumably in response to TNF-α overproduction [13,14]. However, such IL-10 induction may be insufficient to regulate proinflammatory cytokine expression in RA, because the addition of exogenous IL-10 to ST cell cultures markedly reduced TNF-α and IL-1 production [13,15]. These findings suggested the possibility of its therapeutic application in this inflammatory arthritis [16].

In various animal models of arthritis, IL-10 reduced joint swelling, cellular infiltration, cytokine production, and cartilage degradation when administered to animals either before or after induction of disease [16,17]. However, clinical studies performed so far have shown that human recombinant IL-10 (rIL-10) has little therapeutic efficacy in patients with RA [16-18]. Accordingly, immunohistochemical analysis of serial synovial biopsies from the patients treated with IL-10 showed no significant change in inflammatory cell infiltration and expression of TNF-α, IL-1β, and IL-6 after treatment [19]. Thus, IL-10 appears to play a dual role as inhibitor and stimulator in human joint inflammation. In fact, the expression of Fcγ receptor type I (FcγRI; CD64) and FcγRII (CD32) on circulating monocytes was enhanced after IL-10 treatment in patients with RA, and the *in vitro *study showed that IL-10-primed monocytes with high-level expression of FcγRI and FcγRII are able to produce TNF-α in response to immune complexes [18]. In addition, IL-10 stimulates cell surface expression of TNFR2 on RA synovial fluid macrophages, and it enhances the TNF-α effect on IL-1β production by monocytes by increasing surface receptor levels [20]. These findings indicate that IL-10 may contribute to monocyte differentiation into the proinflammatory type of macrophages characteristic of RA.

It has been shown that IL-10 augments the macrophage colony-stimulating factor (M-CSF)-induced growth and differentiation of human monocytes, and macrophages generated in that manner show reactive oxygen intermediate and IL-6 production and Fcγ-mediated phagocytosis more prominently than macrophages generated by M-CSF alone [21]. We have previously shown that CD16 (FcγRIIIA)-expressing monocytes and ST macrophages with high expression of Toll-like receptor (TLR) 2 may be induced by IL-10 and M-CSF and that their TNF-α production could be stimulated by endogenous ligands such as Hsp 60 and immune complexes in RA joints [22,23]. To elucidate the role of IL-10 in monocyte differentiation into TNF-α-responsive tissue macrophages, we investigated the expression and regulation of receptors for IL-10, M-CSF, and TNF-α in blood monocytes and ST macrophages from patients with RA.

## Materials and methods

### Patients and samples

The total patient population consisted of 30 patients with RA (24 women and 6 men; mean ± standard deviation age, 57.2 ± 13.6 years) diagnosed according to the revised 1987 criteria of the American College of Rheumatology (formerly, the American Rheumatism Association) [24]. Patients were receiving prednisolone (≤ 7.5 mg/day; *n *= 22) and disease-modifying antirheumatic drugs such as methotrexate (*n *= 12) and salazosulfapyridine (*n *= 8). They were divided into groups with low, moderate, and high disease activity using the 28-joint disease activity score (DAS28) system [25]; the demographic and laboratory data in these three groups are shown in Table 1. Twenty-six healthy volunteers (20 women and 6 men) matched for age (58.8 ± 15.5 years) served as controls. ST samples were obtained from five patients with RA (4 women and 1 man; age 61.6 ± 3.5 years; disease duration 14.0 ± 2.0 years) at the time of total knee joint replacement. Their clinical parameters were as follows: tender joint count (0–28), 4.2 ± 1.1; swollen joint count (0–28), 2.8 ± 1.5; erythrocyte sedimentation rate, 49 ± 6 mm/hour; serum C-reactive protein, 22 ± 5 mg/liter; immunoglobulin (Ig) M class rheumatoid factor (RF) titer, 61 ± 17 units per ml; visual analogue scale, 40 ± 3 mm; and DAS28, 4.7 ± 0.1. All patients gave informed consent.

### Flow cytometry

Peripheral blood mononuclear cells (PBMCs) were prepared from heparinised blood samples by centrifugation over Ficoll-Hypaque density gradients (Pharmacia, Uppsala, Sweden). Cells were washed well with RPMI 1640 medium (Invitrogen, Carlsbad, CA, USA) and were resuspended in phosphate-buffered saline (PBS) with 1% heat-inactivated fetal calf serum (FCS) (Invitrogen). Cell surface expression of IL-10R1, IL-10R2, M-CSF receptor (M-CSFR), TNFR1, and TNFR2 was analysed by cell surface staining and flow cytometry as described previously [22,23]. PBMC suspensions were incubated with mouse IgG_1 _anti-IL-10R1 monoclonal antibody (mAb) (R&D Systems, Minneapolis, MN, USA), mouse IgG_1 _anti-IL-10R2 mAb (R&D Systems), rabbit IgG anti-M-CSFR (c-fms) polyclonal Ab (Santa Cruz Biotechnology, Inc., Santa Cruz, CA, USA), mouse IgG_2a _anti-TNFR1 mAb (BD Biosciences, San Jose, CA, USA), fluorescein isothiocyanate (FITC)-conjugated mouse IgG_2a _anti-TNFR2 mAb (Genzyme/Techne, Minneapolis, MN, USA), or isotype-matched control Abs. Cells were washed and then incubated with rhodamine-conjugated goat anti-mouse IgG_1 _mAb (Santa Cruz Biotechnology, Inc.) for anti-IL-10R1 mAb and anti-IL-10R2 mAb, phycoerythrin-conjugated goat anti-rabbit IgG mAb (Santa Cruz Biotechnology, Inc.) for anti-M-CSF Ab, or FITC-conjugated anti-mouse IgG_2a _mAb (Santa Cruz Biotechnology, Inc.) for anti-TNFR1 mAb. After washing, cells were resuspended in 1% FCS/PBS. Analysis was performed on a FACScan flow cytometer (BD Biosciences). The monocytes were specifically analysed by selective gating based on parameters of forward and side light scatter. By flow cytometric analysis of PBMCs with anti-CD14 mAb (Becton, Dickinson and Company, Franklin Lakes, NJ, USA), the scattered light-based monocyte population from three patients with RA and three healthy controls was found to contain more than 93% of CD14^+ ^cells, and there was no significant difference in their CD14^+ ^cell frequencies.

### Isolation and culture of blood monocytes

PBMCs were resuspended at a density of 5 × 10^6 ^cells in culture medium (RPMI 1640 medium supplemented with 25 mM HEPES, 2 mM L-glutamine, 2% nonessential amino acids, 100 U/ml penicillin, and 100 μg/ml streptomycin) with 10% FCS. Monocytes were purified from PBMCs by negative selection using a cocktail of Abs against CD3, CD7, CD16, CD19, CD56, CD123, and glycophorin A (monocyte isolation kit II; Miltenyi Biotec GmbH, Bergisch Gladbach, Germany) according to the manufacturer's instructions. The cell suspensions were adjusted at a density of 5 × 10^5 ^cells per ml in culture medium with 10% FCS and were incubated with or without 20% RA ST cell culture supernatants, 25 ng/ml human rIL-10 (R&D Systems), 50 ng/ml human rM-CSF (R&D Systems), or rIL-10 plus rM-CSF in the wells of 48-well plates (Ultra Low Attachment Clusters; Corning Incorporated, Corning, NY, USA) in a humidified atmosphere containing 5% CO_2_. Three days later, the cells were harvested, and cell surface expression of cytokine receptors was determined by flow cytometric analysis.

In addition, monocytes were preincubated with or without 25 ng/ml rIL-10, 50 ng/ml rM-CSF, or rIL-10 plus rM-CSF, and the cells were washed with culture medium and were incubated at a density of 5 × 10^4 ^cells per 200 μl in 96-well plates (Corning Incorporated) with or without 10 ng/ml human rTNF-α (Sigma-Aldrich, St. Louis, MO, USA), 30 μM N6,2'-O-dibutyryladenosine-3'-5'-cyclic monophosphate sodium (dbcAMP) (BIOMOL International, L.P., Exeter, UK), or rTNF-α plus dbcAMP. Twenty-four hours later, cell-free culture supernatants samples were stored at -30°C until the cytokine assay.

To determine IL-10-induced mRNA expression of cytokine receptors, PBMC suspensions were incubated in six-well plates (Corning Incorporated) at 37°C for 2 hours, and adherent monocytes, after removal of nonadherent cells, were gently harvested using a rubber spatula; the purity of monocytes was found to be more than 95%, as determined by flow cytometric analysis of CD14 and CD3 expression. The cells suspensions, adjusted at a density of 5 × 10^5 ^cells per ml in culture medium with 10% FCS, were incubated with or without 25 ng/ml rIL-10. Twelve hours later, the cells were immediately lysed using a reagent for RNA isolation (TRIzol reagent; Invitrogen) and were stored at -80°C until RNA isolation.

RA ST cell culture supernatants were prepared as previously described [26]. Briefly, ST samples from patients with RA were fragmented and digested with collagenase and DNase for 1 hour at 37°C. After tissue debris was removed, cells were resuspended in culture medium with 10% FCS. RA ST cells were incubated at a density of 1 × 10^6 ^cells per ml in 10% FCS/culture medium in six-well plates; 72 hours later, culture supernatants were harvested and stored at -30°C.

### RNA isolation and real-time polymerase chain reaction

Total cellular RNA was extracted from adhered monocytes lysed in TRIzol reagent according to the manufacturer's instructions. Complementary DNA (cDNA) was synthesised from total RNA with Molony murine leukemia virus reverse transcriptase (USB Corporation, Cleveland, OH, USA) and oligo-(dT)_15 _primers (Promega Corporation, Madison, WI, USA). To semiquantify the amounts of TNFR1 and TNFR2 mRNAs, real-time polymerase chain reaction (PCR) was performed with the LightCycler Instrument (Roche Diagnostics, Penzberg, Germany) in glass capillaries. The reaction mix containing DNA double-strand-specific SYBR green I dye (Roche Diagnostics) and specific primers for human TNFR1 and TNFR2 (Nihon Gene Research Laboratories, Sendai, Japan) and β-actin (Search-LC GmbH, Heidelberg, Germany) were added to cDNA dilutions. The cDNA samples were amplified and analysed according to the manufacture's instructions. TNFR1 and TNFR2 expression was determined by normalisation against β-actin expression. The sequences of oligonucleotide primers were as follows: for TNFR1, 5' primer GGT GAC TGT CCC AAC TTT GC and 3' primer GGG TCA TCA GTG TCT AGG CT 3'; for TNFR2, 5' primer CTT CGC TCT TCC AGT TGG and 3' primer AGG CAA GTG AGG CAC CTT.

### Immunoassays for IL-1β and IL-6

Concentrations of IL-1β and IL-6 in monocyte culture supernatants were measured in triplicate by the quantitative sandwich enzyme-linked immunosorbent assay (ELISA) using cytokine-specific capture and biotinylated detection mAbs and recombinant cytokine proteins (all from BD Biosciences) according to the manufacturer's protocol. The detection limits for these cytokines were 7.8 pg/ml.

### Two-color immunofluorescence labeling

Cryostat sections (4 μm) from RA ST samples were fixed in acetone and blocked with 10% rabbit or goat serum for 30 minutes. Double immunofluorescence was performed by serially incubating sections with 10 μg/ml of mouse IgG_1 _anti-IL-10R1 mAb, rabbit IgG anti-TNFR1 Ab, rabbit IgG anti-TNFR2 Ab (Santa Cruz Biotechnology, Inc.), rabbit IgG anti-M-CSFR Ab, mouse IgG_1 _anti-CD68 mAb (BD Biosciences), and isotype-matched control Abs at 4°C overnight, followed by incubation with rhodamine-conjugated goat anti-mouse IgG_1 _mAb (Santa Cruz Biotechnology, Inc.) and FITC-conjugated anti-rabbit IgG mAb (Santa Cruz Biotechnology, Inc.) for 30 minutes at room temperature. The double immunofluorescence of sections was examined with an LSM510 inverted laser-scanning confocal microscope (Carl Zeiss, Jena, Germany) and illuminated with 488 nm and 568 nm of light. Images decorated with FITC and rhodamine were recorded simultaneously through separate optical detectors with a 530 nm band-pass filter and a 590 nm long-pass filter, respectively. Pairs of images were superimposed for colocalisation analysis.

### Microarray analysis of gene expression

IL-10-induced gene expression profiles in monocytes were determined by microarray analysis using the gene expression array kit (SuperArray Bioscience Corporation, Frederick, MD, USA), which detects 367 known genes that encode for inflammatory cytokines, chemokines and their receptors, and cell surface markers, as well as representative signaling proteins and downstream targets of the signal transduction pathways that mediate the immune response. RNA preparation, probe synthesis, hybridisation, chemiluminescent detection, and image and data acquisition and analysis were performed according to the manufacture's protocol. All signal intensities of the genes were normalised to those of β-actin.

### Statistical analysis

Samples with values below the detection limit for the assay were regarded as negative and assigned a value of zero. Data were expressed as the mean ± standard error of the mean. The statistical significance of differences between two groups was determined by the Mann-Whitney *U *test. *P *< 0.05 was considered significant. The correlation coefficient was obtained by Spearman's rank correlation test.

## Results

### Increased expression of IL-10R1 in blood monocytes from patients with active RA

**Figure 1:**
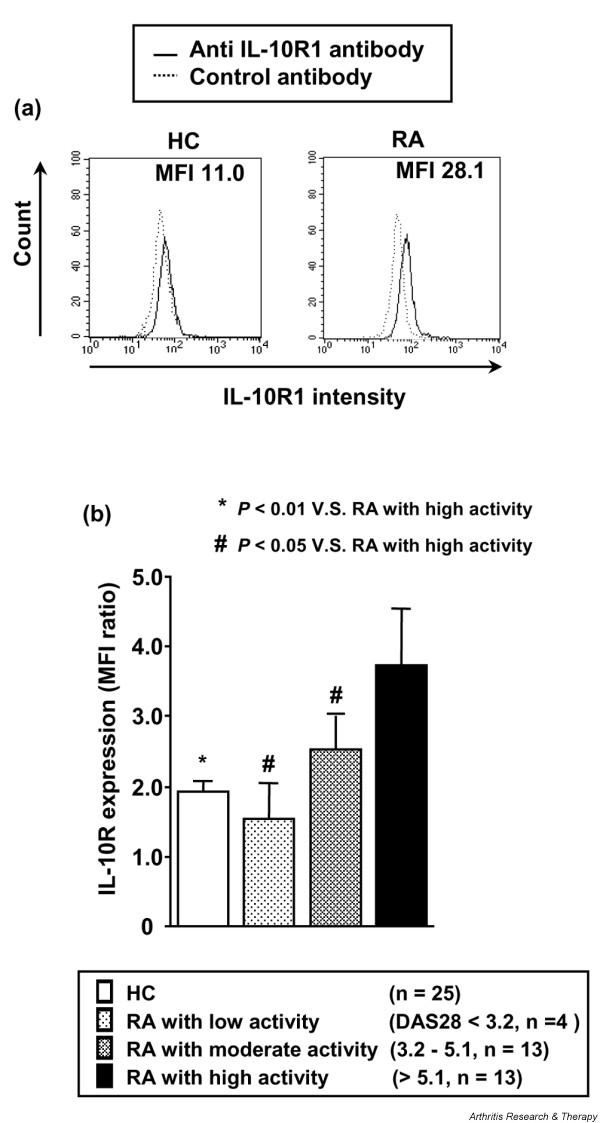
Expression of type 1 interleukin-10 receptor (IL-10R1) on monocytes from patients with rheumatoid arthritis (RA). **(a) **Representative histographic patterns of IL-10R1 expression on monocytes of patients with RA and healthy controls (HCs). Peripheral blood mononuclear cells were stained with mouse immunoglobulin (Ig) G_1 _anti-IL10R1 antibody or isotype-matched control antibody, followed by fluorescein isothiocyanate-conjugated goat anti-mouse IgG_1 _antibody. Flow cytometric analysis of IL-10R1 expression was performed by gating on monocytes according to light scatter profile. **(b) **Increased IL-10R1 expression on monocytes from RA patients with active disease. Patients with RA were divided into groups with low, moderate, and high disease activity according to the disease activity score 28 (DAS28) system. The intensity of cell surface IL-10R1 expression on monocytes from RA patients and HCs was expressed by the ratio of the mean fluorescence intensity (MFI) of staining with anti-IL-10R1 antibody to the MFI of control antibody. Values are the mean ± standard error of the mean. n, number of samples tested.

The IL-10 receptor is a heterodimer consisting of two subunits, and expression of the inducible IL-10R1 seems critical in cellular responses to IL-10 [11]. The cell surface expression of IL-10R1 and IL-10R2 on peripheral blood monocytes from patients with RA and healthy controls was determined by flow cytometric analysis. Figure 1a shows representative histographic patterns of IL-10R1 expression on monocytes. The mean fluorescence intensity (MFI) ratio of IL-10R1 was significantly higher in patients with RA than in controls (RA 2.92 ± 0.42, *n *= 30; controls 1.93 ± 0.16, *n *= 25; *P *< 0.05), but the MFI ratio of IL-10R2 was lower in patients with RA (RA 8.26 ± 0.59; controls 11.81 ± 0.94; *P *< 0.01). To determine the correlation of IL-10R1 levels and disease activity, we divided patients with RA into three groups according to disease activity using the DAS28 system – low activity (DAS28 < 3.2), moderate activity (3.2–5.1), and high activity (>5.1) – and compared the IL-10 R1 intensity of monocytes from these RA groups and controls. As shown in Figure 1b, the levels of IL-10R1 expression incrementally increased in patients with RA with higher disease activity. However, IL-10R1 levels did not correlate with patients' age, disease duration, or the drug therapy employed. These results indicate that RA blood monocytes may become responsive to IL-10 during active disease in terms of receptor expression.

### IL-10R1 induction in monocytes by RA ST cell culture supernatants

**Figure 2:**
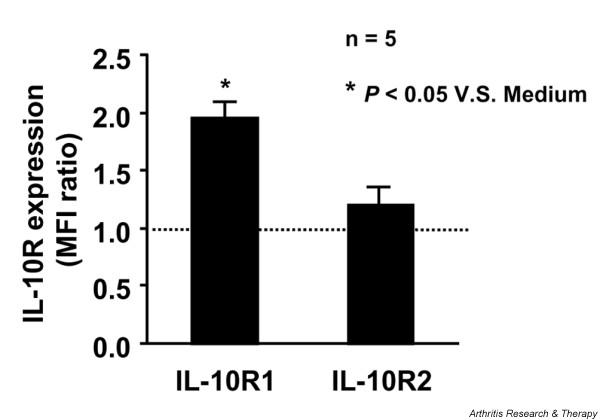
Induction of monocyte expression of type 1 and type 2 interleukin-10 receptor (IL-10R1/2) by culture supernatants of rheumatoid arthritis (RA) synovial tissue (ST) cells. Purified normal monocytes from different individuals (5 × 10^5 ^cells in culture medium with 10% fetal calf serum) were incubated for 3 days with or without a 20% concentration of RA ST cell culture supernatants, and IL-10R1 and IL-10R2 expression was analysed by flow cytometric analysis with anti-IL-10R1, anti-IL-10R2, and control antibodies. The induction of monocyte IL-10R1 and IL-10R2 expression by supernatants was expressed by the ratio of the mean fluorescence intensity (MFI) of IL-10R expression with supernatants to the MFI without supernatants. Values are the mean ± standard error of the mean. n, number of samples tested.

To corroborate the correlation between the high expression of IL-10R1 on monocytes and disease activity, purified monocytes were incubated for 3 days with or without 20% RA ST cell culture supernatants, and the induction of cell surface IL-10R1 and IL-10R2 was determined by flow cytometry. Figure 2 shows the ratio of the MFI of IL-10R1 and IL-10R2 expression with culture supernatants to the MFI with culture medium alone. The levels of IL-10R1, but not IL-10R2, were significantly augmented in the presence of culture supernatants. These results indicate that the increased IL-10R1 on monocytes found in active RA may be induced by a spillover effect of the particular stimuli produced in the inflamed joint, such as cytokines.

### Expression of TNFR1, TNFR2, and M-CSFR in RA blood monocytes

**Figure 3:**
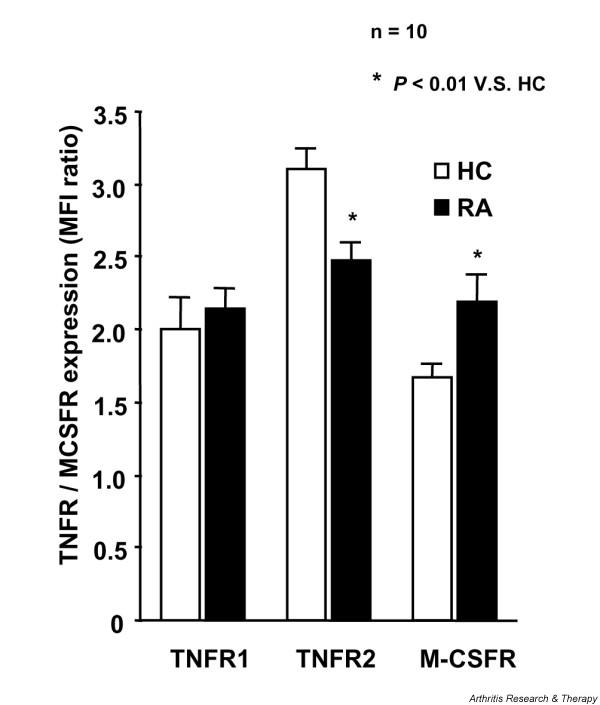
Expression of type 1 and type 2 tumour necrosis factor receptor (TNFR1/2) and macrophage colony-stimulating factor receptor (M-CSFR) on monocytes of patients with rheumatoid arthritis (RA) and healthy controls (HCs). Peripheral blood mononuclear cells were stained with immunoglobulin (Ig) G_2a _anti-TNFR1 antibody, rabbit IgG anti-M-CSFR polyclonal antibody, or isotype-matched control antibody, followed by fluorescein isothiocyanate (FITC)-conjugated anti-mouse IgG_2a _antibody or phycoerythrin-conjugated anti-rabbit IgG antibody, and with FITC-conjugated anti-TNFR2 antibody. TNFR1, TNFR2, and M-CSFR expression on monocytes from patients with RA and HCs was analysed by flow cytometric analysis. The intensity of cytokine receptor was expressed by the ratio of the mean fluorescence intensity (MFI) of staining with anti-cytokine receptor antibody to the MFI of control antibody. Values are the mean ± standard error of the mean. n, number of samples tested.

To examine whether the expression of two distinct receptors for TNF-α, TNFR1 and TNFR2, and of M-CSFR was also increased in RA monocytes, the cell surface expression of these receptors on blood monocytes from patients with RA and healthy controls was determined by flow cytometry. As shown in Figure 3, there was no significant difference in TNFR1 expression between patients with RA and controls; however, TNFR2 expression was lower and M-CSFR expression was higher in the patients. Therefore, RA monocytes appear to partially mature into the cells with high levels of IL-10R1 and M-CSFR expression while in the blood circulation.

### TNFR1 and TNFR2 induction in monocytes by IL-10 and M-CSF

**Figure 4:**
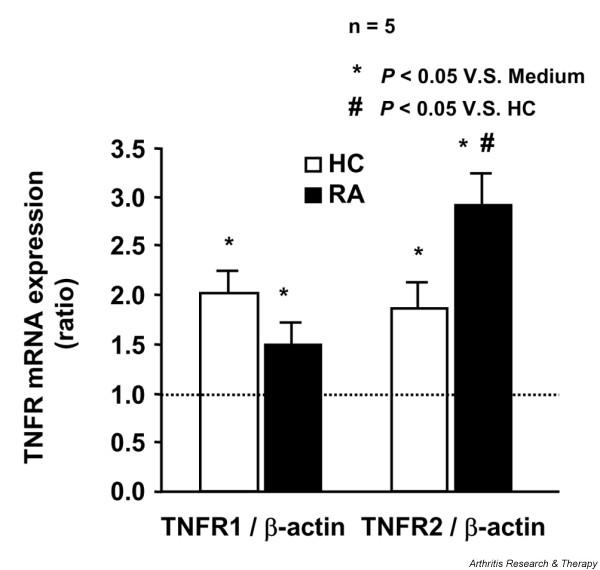
Induction of mRNA expression of type 1 and type 2 tumour necrosis factor receptor (TNFR1/2) in monocytes by interleukin-10 (IL-10). Adhered monocytes from patients with rheumatoid arthritis (RA) and healthy controls (HCs) (5 × 10^5 ^cells in culture medium with 10% fetal calf serum) were incubated for 12 hours with or without 25 ng/ml IL-10. Total cellular RNA was extracted from these monocytes, and mRNA expression was analysed by real-time polymerase chain reaction analysis as described in Materials and methods. Levels of TNFR1 and TNFR2 mRNAs were normalised relative to β-actin expression, and the ratio of the levels of monocytes incubated with IL-10 to the levels without IL-10 was determined. Values are the mean ± standard error of the mean. n, number of samples tested.

Previous studies have demonstrated that IL-10 stimulates cell surface expression of both TNF receptors, in particular TNFR2 on monocytes [20]. To confirm this finding, monocytes from patients with RA and controls were incubated for 12 hours, and TNFR1 and TNFR2 mRNA expression was measured by real-time PCR. As shown in Figure 4, expression of both TNFR receptors was augmented at the transcriptional level by IL-10. RA monocytes were more responsive to IL-10 in terms of TNFR2 induction than were normal monocytes, although TNFR1 induction was weaker in RA monocytes.

**Figure 5:**
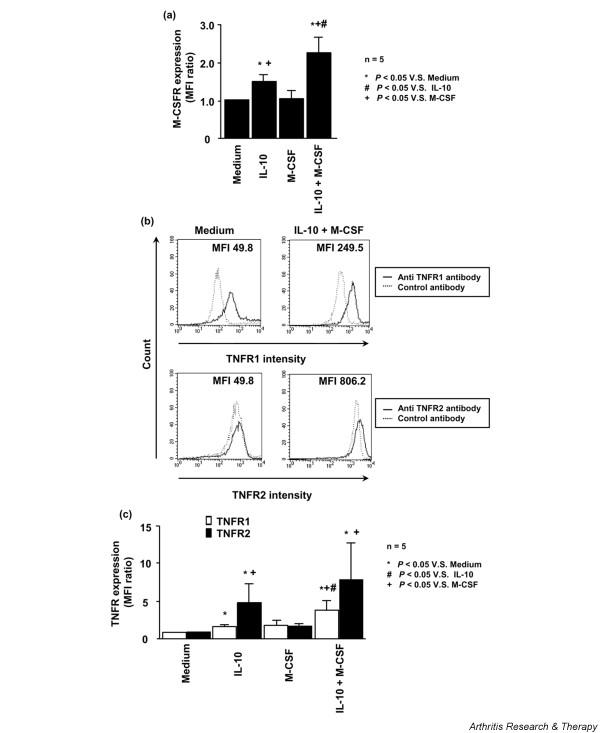
Cytokine receptor induction on monocytes stimulated with interleukin-10 (IL-10) and macrophage colony-stimulating factor (M-CSF). **(a) **Induction of monocyte expression of M-CSF receptor (M-CSFR) by IL-10 and/or M-CSF. Purified normal monocytes from different individuals (5 × 10^5 ^cells in culture medium with 10% fetal calf serum) were incubated for 3 days with or without 50 ng/ml M-CSF, 25 ng/ml IL-10, or IL-10 plus M-CSF. M-CSFR expression was analysed by flow cytometric analysis, and the cytokine-mediated M-CSFR induction was expressed by the ratio of the mean fluorescence intensity (MFI) of M-CSFR expression with cytokines to the MFI without cytokines. Values are the mean ± standard error of the mean. **(b) **Representative histographic patterns of type 1 and type 2 tumour necrosis factor receptor (TNFR1/2) expression on monocytes incubated with or without IL-10 plus M-CSF. TNFR1 and TNFR2 expression was analysed by flow cytometric analysis. **(c) **Induction of monocyte expression of TNFR1 and TNFR2 by IL-10 and/or M-CSF. The cytokine-mediated TNFR1/2 induction was expressed by the ratio of the mean fluorescence intensity (MFI) of TNFR1/2 expression with cytokines to the MFI without cytokines. n, number of samples tested.

IL-10 has been shown to enhance M-CSF-induced macrophage differentiation in part by M-CSFR upregulation [21]. Normal monocytes were incubated for 3 days with or without M-CSF, IL-10, or IL-10 plus M-CSF, and their expression of M-CSF and TNF receptors was analysed by flow cytometry. M-CSFR expression was increased by IL-10 and, more significantly, by IL-10 and M-CSF (Figure 5a). Accordingly, both TNFR1 and TNFR2 expression was increased by IL-10 alone, which was enhanced in the presence of M-CSF (Figure 5b,c). These results indicate that cell surface expression of TNF receptors on monocytes/macrophages may be effectively increased by a combination of IL-10 and M-CSF.

### TNF-α-induced cytokine production in monocytes preincubated with IL-10 and M-CSF

Preliminary culture experiments showed that TNF-α alone failed to stimulate cytokine production by adhered monocytes. In agreement with this finding, previous studies have demonstrated that TNF-α could induce IL-1β production by monocytes when intracellular cAMP levels were elevated by prostaglandin or in the presence of the cAMP analogue dbcAMP and have proved a crucial role of cAMP signaling in the activation of IL-1β gene transcription by site-directed mutagenesis analysis of a CRE (cAMP response element) site in the IL-1β promoter [27].

**Figure 6:**
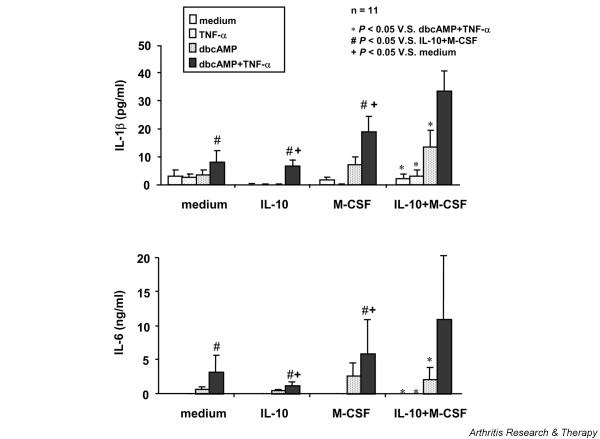
Tumour necrosis factor-α (TNF-α)-mediated interleukin (IL)-1β and IL-6 production by normal monocytes pretreated with IL-10 and/or macrophage colony-stimulating factor (M-CSF). Purified normal monocytes from different individuals were preincubated for 3 days with or without 50 ng/ml M-CSF, 25 ng/ml IL-10, or IL-10 plus M-CSF and then stimulated for 24 hours with or without 10 ng/ml TNF-α, 30 μM dibutyryl cAMP (dbcAMP), or TNF-α plus dbcAMP. Concentrations of IL-1β and IL-6 in culture supernatants were measured in triplicate by the quantitative sandwich enzyme-linked immunosorbent assay. Values are the mean ± standard error of the mean. n, number of samples tested.

To assess the functional significance of increased TNF receptor expression induced by IL-10 and M-CSF, normal monocytes were preincubated for 3 days with or without M-CSF, IL-10, or IL-10 plus M-CSF, then incubated for 24 hours with or without TNF-α stimulation in the presence or absence of dbcAMP, and their IL-1β and IL-6 production was measured by ELISA. In the absence of dbcAMP, adhered monocytes were unable to produce detectable amounts of cytokines in response to TNF-α. Although the stimulatory effect of dbcAMP alone was limited at a concentration of 30 μM, the combination of TNF-α and dbcAMP induced IL-1β and IL-6 production by monocytes preincubated with M-CSF and, more prominently, by monocytes with IL-10 and M-CSF (Figure 6). However, such cytokine production was rather diminished when monocytes were preincubated with IL-10. These results suggest that IL-10, despite its inhibitory potential, may facilitate monocyte differentiation into TNF-α-responsive macrophages in the presence of M-CSF by increasing surface TNF receptor expression.

### Expression of IL-10R1, M-CSFR, TNFR1, and TNFR2 in RA synovial lining macrophages

**Figure 7:**
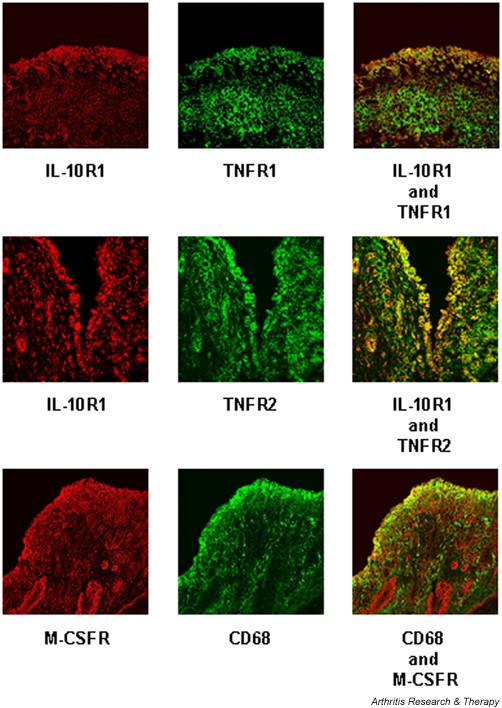
Expression of type 1 interleukin-10 receptor (IL-10R1), macrophage colony-stimulating factor receptor (M-CSFR), and type 1 and 2 tumour necrosis factor receptor (TNFR1/2) in synovial tissue (ST) from patients with rheumatoid arthritis. ST sections were stained with mouse immunoglobulin (Ig) G_1 _anti-IL-10R1 monoclonal antibody (mAb), rabbit IgG anti-TNFR1 Ab, rabbit IgG anti-TNFR2 Ab, rabbit IgG anti-M-CSFR Ab, mouse IgG_1 _anti-CD68 mAb, and isotype-matched control Abs, followed by incubation with rhodamine-conjugated goat anti-mouse IgG_1 _mAb and fluorescein isothiocyanate-conjugated anti-rabbit IgG mAb. Two-color immunofluorescence confocal images were obtained for expression of IL-10R1 (red staining), M-CSFR (red), TNFR1 (green), TNFR2 (green), and CD68 (green). The two images were superimposed, and double-positive cells are shown in yellow. Similar staining patterns were obtained in additional analyses from five ST samples from different patients.

High levels of IL-10, M-CSF, and TNF-α expression have all been detected in the inflamed joint of RA [14,23,28,29]. To confirm the interaction of these cytokines in macrophage differentiation at the site of inflammation, ST samples from five patients with RA were analysed by two-color immunofluorescence labeling using Abs against IL-10R1, M-CSFR, and TNF receptors, as well as anti-CD68 Ab. Figure 7 shows representative staining patterns in RA ST. The majority of M-CSFR^+ ^and IL-10R1^+ ^cells were localised to the lining layer and the endothelial cells, whereas TNFR1^+ ^and TNFR2^+ ^cells were more widely distributed throughout the tissue, including the lining layer and lymphocyte infiltrates. Colocalisation analysis revealed that the CD68^+ ^synovial lining layer contained a large number of cells stained intensely for IL-10R1, M-CSFR, and both TNF receptors, but such cells were sparsely distributed in the sublining layer. Coexpression of these cytokine receptors in lining macrophages suggests that costimulation with IL-10 and M-CSF signals may be involved in the high expression of TNF receptor in lining macrophages in the inflamed ST of RA.

### Microarray analysis of IL-10-induced gene expression in RA monocytes

To further elucidate the roles of IL-10 in monocyte maturation, blood monocytes from two patients with RA were incubated for 12 hours with or without IL-10 and the gene expression profile was analysed by an microarray that detects 367 known genes that encode for cytokines, chemokines and their receptors, cell surface markers, representative signaling proteins, and downstream targets of the signal transduction pathway. Seventeen genes were identified as IL-10-inducible (> twofold increase in gene expression) in both patients with RA, although a total of 87 gene signals were increased by IL-10 in either patient. As shown in Table 2, these genes include the TNF receptor superfamily, chemokine receptors, growth factor receptors, TLRs, and TNF receptor-associated factors (TRAFs), although it also induced the endogenous JAK (Janus kinase) kinase inhibitor SOCS (suppressor of cytokine signaling) 3, as has previously been described [30]. Thus, IL-10 can activate various genes essential for macrophage functions.

## Discussion

IL-10, known as a potent inhibitor for the synthesis of proinflammatory cytokines in monocytes/macrophages and T cells [11], is substantially expressed by lining macrophages and infiltrating T cells in the inflamed ST of RA [13,14]. Studies of RA ST cell cultures indicated that IL-10 may be relatively deficient as compared with proinflammatory cytokines in the joint [13,15], and IL-10 has been protective in animal models of arthritis [16,17], which suggested its therapeutic potential in human RA. However, clinical studies using human rIL-10 have so far failed to prove its potent anti-inflammatory effects in patients with RA [16-18]. Thus, IL-10 may be an important participant of the complex cytokine network of RA, also playing a proinflammatory role in joint inflammation.

In the present study, we demonstrated that blood monocytes from patients with RA express high levels of IL-10R1 and M-CSFR, but not of TNF receptors, and that their TNFR1 and TNFR2 expression is effectively augmented by a combination of IL-10 and M-CSF. Monocytes preincubated with IL-10 and M-CSF are able to respond to TNF-α stimulation by producing IL-1β and IL-6. IL-10R1, M-CSFR, and both types of TNF receptor are all intensively expressed in CD68^+ ^macrophages localised to the lining layer in RA ST. These results suggest that IL-10 may contribute to M-CSF-induced monocyte differentiation into the proinflammatory type of macrophages by increasing TNF receptors in RA, which is thought to partly explain the poor clinical efficacy of IL-10 therapy in the patients.

In association with joint inflammation, blood monocytes become activated before entry into the joint in RA. We have previously demonstrated that CD16-expressing mature monocytes – a subpopulation of cells that, while in the circulation, has acquired features in common with proinflammatory tissue macrophages – are expanded in patients with active disease. CD16^+ ^monocytes can rapidly migrate to the site of inflammation through high-level expression of adhesion molecules and chemokine receptors. The maturation of CD16^+ ^monocytes could be induced by a spillover effect of cytokines produced in the inflamed ST, most notably M-CSF and IL-10 [22,23]. IL-10R1 expression plays a critical role in cellular responses to IL-10 [31]. We found that IL-10R1 expression on monocytes was increased according to the disease activity in patients with RA, as defined by the DAS system, and that the IL-10R1 expression was augmented in the presence of RA ST cell culture supernatants, which indicates that IL-10R1 upregulation may be associated with monocyte maturation induced by RA synovial inflammation. In addition, M-CSFR expression was found to be increased in RA monocytes. Therefore, such activated expression of both IL-10R1 and M-CSFR appears to be involved in CD16^+ ^monocyte maturation.

M-CSF, although originally identified as a hematopoietic growth factor of macrophage-lineage cells, stimulates the survival, proliferation, and differentiation into macrophages and osteoclasts of monocytes and activates their functions such as cytokine production [32]. The involvement of M-CSF in RA pathogenesis has been suggested by *in vivo *studies of collagen-induced arthritis [33]. In this murine model of autoimmune arthritis, M-CSF administration exacerbated disease symptoms, neutralisation of endogenous M-CSF with Ab reduced the disease severity, and M-CSF-deficient mice (*op/op*) showed no chronic arthritis. In RA joints, high levels of M-CSF have been detected [23,34,35], and the cellular source of this proinflammatory cytokine includes synovial fibroblasts, chondrocytes, and tissue macrophages [34,36-38]. In particular, synovial fibroblasts constitutively produce M-CSF, and their production is believed to be markedly increased in the cytokine milieu, where potent stimulators such as TNF-α and IL-1 are abundant [1,36,38].

Higher levels of IL-10R1 and M-CSFR were expressed on monocytes from patients with RA as compared with healthy controls, but their expression of both TNFR1 and TNFR2 was not increased. By two-color immunofluorescence labeling of RA ST samples, synovial lining macrophages were found to strongly express both types of TNF receptor, as well as IL-10R1 and M-CSFR. Macrophages accumulating in the lining layer represent the highly activated phenotype of this lineage, as evidenced by high levels of cell surface expression of CD16, CD68, major histocompatibility complex class II antigens, and various adhesion molecules, and by overproduction of the essential proinflammatory cytokines TNF-α and IL-1β [39]. We found that expression of both TNF receptors in monocytes was increased by IL-10 stimulation, and more efficiently in combination with M-CSF, and that *in vitro *differentiated macrophages in the presence of IL-10 and M-CSF could respond to TNF-α stimulation by inducing other cytokines such as IL-1β and IL-6. Therefore, monocytes with high-level expression of IL-10R1 and M-CSFR may readily differentiate into TNF-α-responsive macrophages in the inflamed joint, where these receptor ligands are produced at high levels. In this regard, RA monocytes are thought to be undergoing maturation into macrophages before entry into the joints.

IL-10 may not be a general inhibitor of inflammatory responses in RA, but rather a stimulator in terms of monocyte differentiation. In addition to its induction of TNF receptors, IL-10 has been shown to potentiate immune complex-mediated proinflammatory responses and tissue destruction by stimulating FcγRI and FcγRII expression on monocytes [18]. Such IL-10 induction of inflammatory molecules in monocytes/macrophages was confirmed by our microarray analysis of gene expression showing that IL-10 activated various genes essential for macrophage functions, including other members of the TNF receptor superfamily, receptors for chemokines and growth factors, TLRs, and TRAFs. Furthermore, most of the IL-10 effects on B-cell function are stimulatory. IL-10 has been implicated in the maturation of B cells into plasma cells in the presence of synovial fibroblasts from RA [40], the spontaneous IgM-RF production by B cells [41], and the Th2 cell-mediated B-cell Ig production [29]. These B-cell stimulatory effects of IL-10 could be important in the perpetuation of RA inflammation because an inflammatory role of B cells in the pathogenesis has recently been supported by clinical efficacy of B-cell depletion therapy with anti-CD20 Ab in patients with RA resistant to TNF-α inhibitors [42]. It would therefore seem that the positive effects of IL-10 on macrophage and B-cell maturation may neutralise its otherwise anti-inflammatory properties in RA, as has been shown by clinical studies [17,18].

## Conclusion

Blood monocytes from patients with RA express high levels of IL-10R1 and M-CSFR in association with joint inflammation, and their TNFR1 and TNFR2 expression is effectively augmented by a combination of IL-10 and M-CSF. Monocytes preincubated with IL-10 and M-CSF are able to respond to TNF-α stimulation by producing IL-1β and IL-6. Receptors for IL-10, M-CSF, and TNF-α are all intensively expressed by lining CD68^+ ^macrophages in the ST lesion of RA. Therefore, IL-10 may play an important role in M-CSF-induced monocyte differentiation into TNF-α-responsive macrophages by increasing TNF receptors in RA joints.

## Abbreviations

cDNA = complementary DNA; DAS = disease activity score; dbcAMP = N6,2'-O-dibutyryladenosine-3'-5'-cyclic monophosphate sodium; ELISA = enzyme-linked immunosorbent assay; FcγRI = Fcγ receptor type I; FCS = fetal calf serum; FITC = fluorescein isothiocyanate; Ig = immunoglobulin; IL = interleukin; IL-10R1/2 = type 1 and 2 interleukin-10 receptor; mAb = monoclonal antibody; M-CSF = macrophage-colony stimulating factor; M-CSFR = macrophage-colony stimulating factor receptor; MFI = mean fluorescence intensity; NF = nuclear factor; PBMC = peripheral blood mononuclear cell; PBS = phosphate-buffered saline; PCR = polymerase chain reaction; RA = rheumatoid arthritis; RF = rheumatoid factor; ST = synovial tissue; TLR = Toll-like receptor; TNF-α = tumour necrosis factor-α; TNFR1/2 = type 1 and 2 tumour necrosis factor receptor; TRAF = tumour necrosis factor receptor-associated factor.

## Competing interests

The authors declare that they have no competing interests.

## Authors' contributions

KT was responsible for the experiments and data analysis and wrote the report. M Yamamura was responsible for the planning of the research and wrote up the manuscript. MI, FO, JY, KS, and MK assisted the experiments. M Yamada and HM contributed to the planning of the research. All authors read and approved the final manuscript.

## Figures and Tables

**Table 1:** Demographic and clinical data of patients with rheumatoid arthritis

	Low activity^a ^(*n *= 4)	Moderate activity (*n *= 13)	High activity (*n *= 13)
Age (years)	55.2 ± 8.0^b^	55.0 ± 4.5	59.1 ± 2.9
Gender (men/women)	1/3	3/10	2/11
Disease duration (years)	1.3 ± 0.2	6.6 ± 1.4	4.5 ± 3.0
Tender joint count	0.3 ± 0.3	2.5 ± 0.5	10.0 ± 2.2
Swollen joint count	0.0 ± 0.0	3.2 ± 0.9	10.5 ± 2.0
ESR (mm/hour)	21.5 ± 7.3	43.5 ± 6.0	67.7 ± 8.7
CRP (mg/liter)	10.1 ± 6.9	9.2 ± 1.8	47.7 ± 9.3
Rheumatoid factor (units/ml)	145 ± 106	138 ± 53	203 ± 62
Visual analogue scale (mm)	8 ± 5	30 ± 6	57 ± 7
DAS28	2.3 ± 0.4	4.2 ± 0.2	6.1 ± 0.2

^a^Divided into patient groups with low (<3.2), moderate (3.2–5.1), and high disease activity (>5.1) according to the 28-joint disease activity score (DAS28). ^b^Mean ± standard error. CRP, C-reactive protein; ESR, erythrocyte sedimentation rate.

**Table 2:** Major interleukin-10 (IL-10)-induced transcripts in monocytes from patients with rheumatoid arthritis

Symbol	Encoded protein	Gene name	IL-10-induced changes (folds)	
			
			Patient 1	Patient 2
BMPR1A	Bone morphogenetic protein receptor, type IA	*ALK-3*	2.41	2.37
BMPR1B	Bone morphogenetic protein receptor, type IB	*ALK-6*	0.74	9.79
BMPR2	Bone morphogenetic protein receptor, type II	*BMPR-II*	1.81	1.32
CCL15	Chemokine (C-C motif) ligand 15	*MIP-1 delta/MIP-5*	1.84	1.07
CCR5	Chemokine (C-C motif) receptor 5	*CCR5*	1.93	1.11
CXCL3	Chemokine (C-X-C motif) ligand 3	*GRO3*	2.17	0.83
IRAK2	Interleukin-1 receptor-associated kinase 2	*IRAK2*	1.93	2.73
SOCS3	Suppressor of cytokine signaling 3	*SSI-3*	3.84	15.29
TGFBR2	Transforming growth factor, β receptor II	*TGFBR2*	2.17	0.79
TGFBR3	Transforming growth factor, β receptor III	*TGFBR3*	2.04	0.29
TIMP1	Tissue inhibitor of metalloproteinase 1	*TIMP1*	3.49	1.46
TLR2	*Homo sapiens *Toll-like receptor 2	*TLR2*	1.38	2.28
TLR3	*Homo sapiens *Toll-like receptor 3	*TLR3*	3.03	16.61
TLR4	*Homo sapiens *Toll-like receptor 4	*TLR4*	2.66	1.86
TLR9	*Homo sapiens *Toll-like receptor 9	*TLR9*	2.44	0.78
TLR10	*Homo sapiens *Toll-like receptor 10	*TLR10*	2.78	0.90
TNFRSF1B	Tumour necrosis factor receptor superfamily, member 1B	*TNFR2/p75*	2.64	0.91
TNFRSF7	Tumour necrosis factor receptor superfamily, member 7	*CD27*	8.99	0.57
TNFRSF8	Tumour necrosis factor receptor superfamily, member 8	*CD30*	3.13	1.15
TNFSF11	Tumour necrosis factor (ligand) superfamily, member 11	*TRANCE*	3.50	2.01
TNFRSF11A	Tumour necrosis factor receptor superfamily, member 11a, activator of nuclear factor-κB	*Rank*	2.50	0.56
TNFSF4	Tumour necrosis factor (ligand) superfamily, member 4	*OX40L*	2.67	1.06
TRAF4	TNF receptor-associated factor 4	*TRAF4*	2.18	1.00
TRAF6	TNF receptor-associated factor 6	*TRAF6*	3.49	0.56
PUC18	PUC18 plasmid DNA	*pUC18*	0.00	0.00
ACTB	β-actin	*β-actin*	0.92	0.91
